# Linear Viscoelastic Wood Creep Models

**DOI:** 10.3390/ma18235348

**Published:** 2025-11-27

**Authors:** Tomasz Socha, Krzysztof Kula, Arkadiusz Denisiewicz

**Affiliations:** Institute of Civil Engineering, Faculty of Engineering and Technical Sciences, University of Zielona Góra, ul. Prof. Z. Szafrana 1, PL-65516 Zielona Góra, Poland; k.kula@ib.uz.zgora.pl (K.K.); a.denisiewicz@ib.uz.zgora.pl (A.D.)

**Keywords:** experimental verification, long-term multistage loading, model identification, pine (*Pinus sylvestris*) wood beams, six-parameter model, wood rheology

## Abstract

The paper focuses on problems of the rheology of wooden beams. The main aim of the theoretical and experimental research was to select a linear viscoelastic rheological model best describing the behavior of wooden beams. The experimental elements were full-size beams made of pine wood. The beams were subjected to a long-term multistage loading program. Simple models of linear viscoelastic materials, i.e., the three-parameter standard model and the four-parameter Burgers’ model, reveal essential restrictions and are too poor to describe processes of wood creeping, particularly in the initial period of the phenomenon, as well as in the case of multistage loads. Our research has shown that, under constant heat and moisture conditions, the five-parameter rheological model is sufficient to describe wood creep under constant loading. In contrast, the model deviates significantly from experimental results in the case of a multistage loading program. In view of this, a six-parameter rheological model was used, which fits the experimental data perfectly. Thus, the decisive verification of the assumed rheological model can be ensured only by carrying out experiments with the use of a multistage program of loads (clear separation of the identification of the model’s parameters from its verification).

## 1. Introduction

Wood is one of the oldest construction materials used by our civilization, and it continues to be effectively applied in many engineering applications [1,2]. Undoubtedly, the main advantages of wood include its relatively low weight, high strength in compression and tension, and ease of processing and assembly. For example, in the case of pine wood (*Pinus sylvestris*): compressive strength parallel to the grain: approximately 40–50 MPa, tensile strength parallel to the grain: approximately 90–120 MPa, bending strength perpendicular to the grain: typically 2–5 MPa. These values may be reduced due to the presence of various wood defects, particularly knots. The ecological aspect is also significant; wood has a low thermal conductivity coefficient and is a renewable raw material. Due to these properties, this material is currently utilized in structures where steel and reinforced concrete have traditionally dominated. Examples of such structures include bridges [3,4].

Wood, despite its many advantages, also has certain drawbacks. It exhibits several internal structural flaws and is an inhomogeneous and anisotropic material. Its properties depend on various factors such as moisture content, temperature, and long-term loading [5]. Its strength also decreases due to structural defects or improper fiber orientation. Despite its high chemical resistance, it is susceptible to attack by various pests and fungi.

Wood exhibits the properties of a viscoelastic material. For several reasons, this presents a significant engineering and scientific challenge. The rheological creep of wooden elements subjected to long-term loading can exceed the initial elastic strain by multiple times. Conversely, in wooden structures reinforced with other materials, there can be a substantial redistribution of stresses due to the differing rheological parameters of the connected components. Therefore, selecting an appropriate rheological model and accurately identifying its parameters are essential. Furthermore, the chosen rheological model should, on one hand, be sufficiently comprehensive to accurately represent most aspects of wood rheology, and on the other hand, remain simple enough to facilitate practical application in engineering calculations.

Due to the highly inhomogeneous internal structure of wood, and consequently the difficulty in selecting samples with similar properties and structure for testing (a problem especially evident in natural-scale elements), the results of such experiments tend to exhibit significant variations in values even under constant thermal and moisture conditions. An illustration of this can be found in the studies conducted by a team led by Fridley [6]. They measured the deflections of simply supported pine beams with dimensions of 3.0 cm × 18.4 cm × 391.0 cm over a period of 120 days, during which the load was removed at the midpoint halfway through the testing period. The initial elastic deflections ranged from 8.27 mm to 21.34 mm, while the rheological increases in deflection (relative to the initial deflection) varied between 31% and 94%.

Considering the above information, it is not surprising that there is a wide variety of models used by many researchers to describe the rheology of wood. Among the most commonly employed are power-law models, the three-parameter standard model, the four-parameter Burgers’ model, and models built upon these, supplemented with components that describe the behavior of wood under varying thermal and moisture conditions.

The power-law model has been used, among others, by Clouser [7], Gerhards [8], Hoyle [9], Fridley, and Soltis [10,11,12]. The empirical power-law creep model exhibits several drawbacks. Firstly, it can only be applied to describe creep under constant load over time. Secondly, the constants in its equations lack a clear physical meaning.

Empirical power-law models do not suffer from the drawbacks of linearly viscoelastic models constructed from simple Hooke’s and Newton’s elements. The first attempts in this area involved using the three-parameter standard model and the four-parameter Burgers’ model. Research related to the application of these models was carried out by Hoyle, Senft, Suddarth, and Fridley [11,13], as well as Brokans and Ozola [14]. The four-parameter model can be applied for long-term prediction of beam behavior under bending. However, under variable climatic conditions, the behavior of the tested samples was modeled with large discrepancies from the predictions.

Toratti [15] proposed several approaches for modeling the rheology of wood. One of the suggested models consists of seven Kelvin–Voigt bodies connected in series, along with an additional component that accounts for hygroexpansion.

Toratti’s model was used by Schänzlin [16] to analyze the creep behavior of wooden structures built in the southwestern region of Germany. A good agreement with experimental data was achieved. This work’s exceptional value should be highlighted. It represents the most comprehensive current overview of the rheology of wooden structures. The authors have conducted an in-depth analysis of existing models, their validation against experimental data, and the influence of various factors on the rheological behavior of wood. Among the most effective models describing wood rheology is the one proposed by Toratti. This model consists of six Kelvin–Voigt elements for pure creep, a single Kelvin–Voigt element for mechano-sorptive creep, and an additional component accounting for hydroexpansion.

The rheological model developed by Hanhijärvi [17] features nine Maxwell bodies arranged in parallel, with a spring also in parallel to these elements. To each of these parallel sets, a hygro-expansion component is incorporated.

In the model proposed by Becker [18], four Kelvin–Voigt bodies are used to depict the normal creep behavior, along with an additional Kelvin–Voigt element representing mechano-sorptive creep. To account for inelastic strains caused by moisture variations and nonlinear creep, extra components are integrated into the model.

Mårtensson [19] proposed a rheological model composed of eight Kelvin–Voigt elements, along with additional components that account for mechano-sorptive phenomena. The calculations show that the model is capable of describing the response of wood with reasonable accuracy. The simulations indicate that the behavior of small test specimens is more challenging to accurately describe than that of larger beams. Some differences in response are found to depend on the loading mode and the nature of moisture cycling. Very large and rapid moisture cycles appear to cause greater mechano-sorptive effects than smaller variations. The results also demonstrate that strain has a significant influence on the shrinkage and swelling response.

A comprehensive overview of other models used to describe the rheology of wood is presented in the works [20,21,22,23,24,25,26].

The latest research on wood rheology is presented in the work of Naghdinasab et al. [27]. The authors correctly observed that most creep models currently in use are only reliable within the timescale of the available experimental data, which typically covers just a few years. However, predicting long-term behavior—such as over 100 years—requires greater accuracy, an objective that is challenging to achieve experimentally. The study compares several established creep models, including Kelvin–Voigt type models, using various experimental datasets, such as constant climate creep of spruce clearwood and timber. The models are fitted via regression analysis and evaluated based on their number of parameters. Their long-term performance is then extrapolated to assess behavior over a 100-year period. The findings confirm that four-element Kelvin–Voigt models provide the best balance between predictive accuracy and model complexity, making them particularly suitable for long-term predictions.

In order to consider the time-dependent behavior in the structural design of timber elements, creep coefficients are defined by the standards Eurocode 2 [28] and Eurocode 5 [29]. These creep coefficients describe the ratio between creep strain and elastic strain:

(1) $$k_{def}=\frac{\varepsilon_{cr}}{\varepsilon_{el}},$$

where

*ε_cr_*—strain due to creep

*ε_el_*—elastic strain

*k_def_*—creep coefficient

The creep coefficient for solid timber, which depends on the ratio of permanent load *g* to the total load *p*, as well as on the equilibrium moisture content, ranges from 0.5 to 2.0. Due to the varying creep coefficients, in some cases—particularly for small permanent load ratios (*g*/*q* < 0.5)—the slenderness of the cross-sections may be limited by maximum deflection restrictions.

Another issue arises in the design of increasingly popular composite structures made of wood combined with other materials, especially fiber-reinforced polymer tapes and various types of adhesives. Each of these materials exhibits rheological properties. As a result, there will be not only rheological creep but also a significant redistribution of stresses within the cross-section of the composite structure, which can completely alter the structural behavior.

The above information demonstrates that the rheology problems of wood remain unresolved in a satisfactory manner. The rheological models proposed by various authors are highly complex and require the determination of numerous parameters. This makes their practical engineering application significantly challenging. Moreover, nonlinear models pose substantial computational difficulties when dealing with time-varying loads and when analyzing the performance of composite structures composed of multiple materials with different rheological properties (due to the inability to apply the Boltzmann superposition principle [30,31]).

This issue, coupled with the current widespread use of bonded reinforcing composite elements, has served as the foundation for an extensive research program undertaken by the authors. The primary goal was to develop a theoretical model of a multi-layer beam (comprising wood, composite reinforcement, and a thick adhesive layer that also functions as an additional reinforcement) made of materials with rheological properties, and to experimentally validate this model. In such systems, the rheological behavior of the constituent materials can lead to numerous challenges, including significant stress redistribution. Therefore, it is crucial to select an appropriate rheological model for each material, determine the numerical values of its parameters, and validate it experimentally—preferably through a multi-step loading program. The rheological models sought must be sufficiently complex to accurately capture material behavior under varying load levels and during unloading, yet simple enough to be implemented in the multi-layer beam model without introducing insurmountable mathematical difficulties. This includes using relatively straightforward relaxation functions and enabling the application of the Boltzmann superposition principle. Generally, a more sophisticated theoretical model tends to better fit experimental data; however, due to their complexity, such models are often less practical for specific predictive calculations, integration into other mathematical models, or engineering applications.

In accordance with the points outlined above, the authors developed an extensive experimental research program, which included both destructive and rheological tests of full-scale wooden beams reinforced with fiber-reinforced composite tendons, as well as tests on samples of the individual materials (wood, fiber-reinforced composite, and adhesive). This paper presents the results of the timber experiments. As previously stated, the primary goal was to identify the simplest possible linearly viscoplastic rheological model for wood. This model must accurately capture the rheological behavior of wood over varying load durations and magnitudes. Additionally, it should be sufficiently simple mathematically to be integrated into a multi-material rheological model.

The results of the modeling and analysis for various materials (fiber-reinforced rods, adhesive) and the reinforced beams will be explored in subsequent publications by the authors.

## 2. Materials and Methods

### 2.1. Research Setup and Specimens

To experimentally observe the creep phenomenon in wood and to validate the proposed theoretical models, an experimental research program was developed involving full-scale wooden beams using a multistage loading approach.

**Figure 1:**
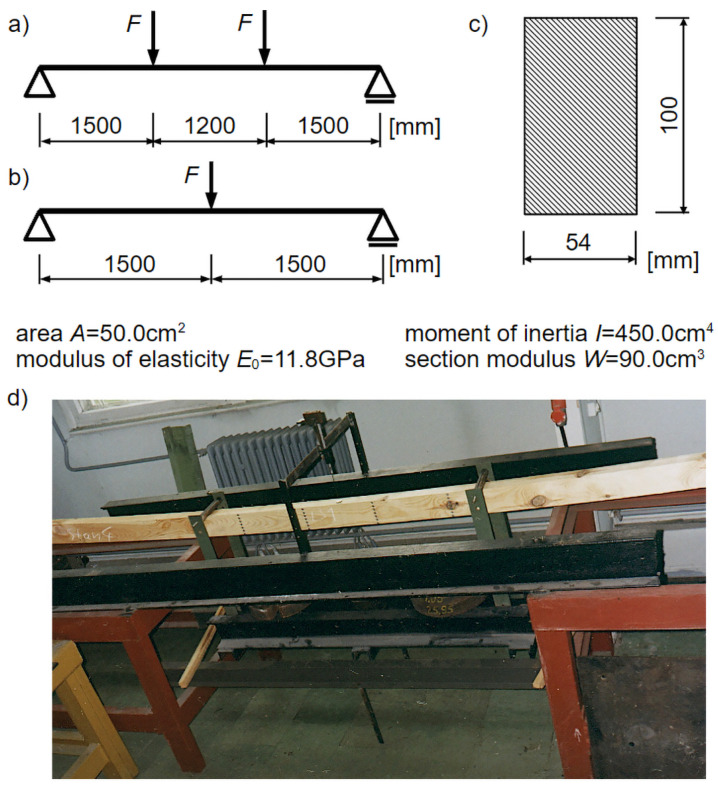
Static scheme and cross-sections of beams: (**a**) static scheme in rheological tests, (**b**) static scheme in destructive tests, (**c**) cross-section of wooden beam, (**d**) one of the beams during testing.

The experimental elements consisted of 12 units: 6 for destructive tests, labeled LN1–LN6, and 6 for rheological tests, labeled LS1–LS6. These were single-span beams made of pine wood (*Pinus sylvestris*) with a rectangular cross-section measuring 54 mm by 100 mm and a span of 4.20 m in rheological tests (Figure 1a). Conversely, destructive tests were conducted using a three-point bending test on beams with the same cross-section and a span of 3.0 m (Figure 1b). These dimensions were primarily determined by laboratory conditions. Literature [32] recommends conducting tests on full-scale elements with a span of approximately 4.50 m between supports, as smaller specimens tend to produce overestimated results. Therefore, the chosen support spacing is considered justified.

Materials with particularly favorable grain orientation or knot arrangements for bending were not selected for further testing. The distribution of wood defects was random. Before starting the experiments, the beams were seasonally dried in the laboratory where the tests were subsequently conducted. Throughout the seasoning and testing periods, the laboratory temperature was maintained between 18 and 22 °C, and the relative humidity was kept within 40% to 60%. These were not fully constant thermal-humidity conditions, but the range of parameter variations was considered to have a minimal impact on the beams at a full-scale level. Temperature and ambient humidity were monitored once a day throughout the entire study period, and if necessary, the operation of heating devices was slightly adjusted.

The tests were conducted in accordance with the requirements [33], which were modified due to the dimensions of the applied beams. For the batch of 6 beams (LN1–LN6), destructive testing was performed to determine the load levels for the rheological experiments. These destructive tests were carried out using a universal strength testing machine, Instron 8804 (Instron Ltd., High Wycombe, UK), equipped with a hydraulic loading system and a specially designed stand.

The results of the destructive tests were used to determine the necessary load levels for the rheological experiments. According to available literature data [34], wood can be considered a linearly viscoelastic material at a load level of approximately 30% of the ultimate load. Therefore, the baseline load was set to produce stresses close to this value $30\%{\bar{\sigma }}_{n}^{L}$, where ${\bar{\sigma }}_{n}^{L}$ is the average stress at the edge of the beam at the moment of failure.

Only linear viscoelastic models of wood rheology were assumed. This approach was necessary due to the requirement to use such a linear model in the theory of rheology for multi-material systems, which the authors employ to describe the test results of wooden beams reinforced with bonded fiber composites—a topic that will be covered in separate publications. Given the considerable variation in wood properties, load levels slightly above and below 30% were also included in the tests. The multi-stage loading and unloading program further enabled more robust validation of the rheological model.

To carry out the rheological testing program, laboratory test setups were prepared, consisting of workbenches with supports for the beams and a loading system using a suspended weight of the required magnitude. The appearance of this setup is shown in Figure 1.

The purpose of the rheological tests under long-term static loads was to determine a linear viscoelastic model of wood and to identify its parameters—specifically, the elastic modulus $E_{j}^{i}$ and the viscosity coefficient $\eta_{j}^{i}$. Considering the need for comprehensive experimental validation of the wood rheology model, a multistage program of long-term static loads was adopted, described in detail below.

Mechanical sensors with a measurement range of 150 mm and an accuracy of 0.02 mm were used to measure deflections. To measure strain increments, a PROCEQ S.A. mechanical strain gauge with a measurement base of 300 mm and an accuracy of 0.001 mm was employed. To perform readings on each beam in the bending zone, steel reference marks were attached (4 columns spaced 30 cm apart, with 7 elements in each column, and vertical distances between reference marks of 1.5 cm), as shown in Figure 1.

Deflection readings were taken daily (at the same time), both during the application of load and after unloading. Strain increments were measured at the following specific moments:

1. Before applying the load and immediately after its application;
2. At 0, 1, 3, 7, 14, 21, 28, 35, 49, and so on, every 14 days, as well as on days 91 and 100 of the load application period;
3. After 100 days, the beams were unloaded, and strain increments were measured immediately before and after unloading, followed by measurements according to the schedule in effect during the load application period;
4. After 35 days, the beams were reloaded, and the strain measurement schedule was repeated.

The results of these measurements, obtained during the first load period (0–100 days), were used to determine the values of the parameters $E_{j}^{i}$ and $\eta_{j}^{i}$ for the tested linear viscoelastic rheological models. Meanwhile, the results obtained at subsequent load and unload levels served to validate these models and select the best one among them. Due to the high ratio of beam span to cross-sectional height l/h=42, the influence of transverse forces on deflections was neglected in further analysis.

The beams were installed on the test stands only once and remained there throughout the entire testing period of 345 days. Only the suspended load was changed. During load changes, deflections were measured immediately before and after the adjustment. Each change lasted about 15 min. Due to the long duration of the experiment and the size of the beams, it was assumed that these deflection measurements were effectively carried out simultaneously.

### 2.2. Theoretical Foundations

The general integral form of the equations for linearly viscoelastic media under uniaxial stress, expressed using the convolution product, is given by the equations [30]:

(2) ε=J∗dσ,

(3) σ=E∗dε.

Rheological deflections of a beam subjected to long-term loads can be determined using the elastoviscoplastic analogy. Any linear viscoelastic problem can be solved similarly to its corresponding linear elasticity problem, with forces and displacements treated as functions of the complex variable *s*. Elastic constants are replaced by appropriate operators. The solution to the viscoelastic problem can be obtained through the inverse Laplace transform of the formulated “fictitious” elastic problem [29]. The basis for calculating the deflection of a viscoelastic beam is therefore the classical elastic solution:

(4) $$\frac{d^{2}u_{0}\left(x,t\right)}{dx^{2}}=-\frac{M\left(x,t\right)}{E_{0}\cdot I}.$$

Applying the Laplace transform to relation (2), we obtain:

(5) $$L\left[\varepsilon \right]=L\left[J*\partial \sigma \right].$$

Using the properties of this transformation, relation (5) can be written as:

(6) $$L\left[\varepsilon \right]=L\left[J\right]\cdot L\left[\partial \sigma \right],$$

(7) $$L\left[\varepsilon \right]=L\left[J\right]\cdot s\cdot L\left[\sigma \right],$$

(8) $$\tilde{\varepsilon }\left(x,s\right)=s\cdot \tilde{J}\left(s\right)\cdot \tilde{\sigma }\left(x,s\right).$$

In the case of neglecting rheological phenomena, the constitutive relation (2) can be reduced to Hooke’s law:

(9) $$\varepsilon \left(x\right)=\frac{\sigma \left(x\right)}{E_{0}}.$$

After applying the Laplace transform to Equation (9), the solution of the “fictitious” elastic problem was obtained:

(10) $$\tilde{\varepsilon }\left(s\right)=\frac{\tilde{\sigma }\left(s\right)}{\hat{E}\left(s\right)},$$

in which this operator $\hat{E}\left(s\right)$ replaces the Young’s modulus $E_{0}$. This operator can be determined by comparing the right-hand sides of Equations (8) and (10):

(11) $$\frac{\tilde{\sigma }\left(x,s\right)}{\hat{E}\left(s\right)}=s\cdot \tilde{J}\left(s\right)\cdot \tilde{\sigma }\left(x,s\right),$$

(12) $$\hat{E}\left(s\right)=\frac{1}{s\cdot \tilde{J}\left(s\right)}.$$

Applying the Laplace transform to Equation (4) yields the relation describing the “fictitious” elastic problem:

(13) $$\frac{d^{2}{\tilde{u}}_{t}\left(x,s\right)}{dx^{2}}=-\frac{1}{I}\cdot \frac{\tilde{M}\left(x,s\right)}{\hat{E}\left(s\right)},$$

and, after considering (12):

(14) $$\frac{d^{2}{\tilde{u}}_{t}\left(x,s\right)}{dx^{2}}=-\frac{1}{I}\cdot s\cdot \tilde{J}\left(s\right)\cdot \tilde{M}\left(x,s\right).$$

The solution to the viscoelastic problem was obtained through the inverse Laplace transform of relation (14):

(15) $$\frac{d^{2}u_{t}\left(x,t\right)}{dx^{2}}=-\frac{1}{I}\cdot J\left(t\right)*dM\left(x,t\right).$$

In the special case of a constant load over time, the relationship between the bending moment and time can be expressed as a product using the Heaviside function *H(t):*

(16) $$M\left(x,t\right)=M\left(x\right)\cdot H\left(t\right).$$

After considering relation (16) and the properties of the convolution product, the Heaviside function *H(t)*, and the Dirac *δ* function:

(17) $$\frac{dH\left(t\right)}{dt}=\delta \left(t\right),$$

(18) f∗δ=f,

Equation (15) can be transformed into the following form:

(19) $$\frac{d^{2}u_{t}\left(x,t\right)}{dx^{2}}=-\frac{1}{I}\cdot J\left(t\right)\cdot M\left(x\right).$$

Elastic and viscoelastic deflections, ($u_{0}$ and $u_{t}$), can be calculated by double integration of Equations (4) and (19):

(20) $$u_{0}=\frac{1}{E_{0}\cdot I}\cdot \left[\int_{x}\;\left(\int_{x}M\;dx+C\right)dx+D\right],$$

(21) $$u_{t}=\frac{J}{I}\cdot \left[\int_{x}\;\left(\int_{x}M\;dx+C\right)dx+D\right].$$

The ratio of these relations is expressed by the equation:

(22) $$\frac{u_{t}}{u_{0}}=E_{0}\cdot J\left(t\right),$$

which simplifies to:

(23) $$u_{t}=u_{0}\cdot E_{0}\cdot J\left(t\right),$$

From Formula (23), it follows that the viscoelastic deflection $u_{t}$ of a beam made from a linearly viscoelastic material can be obtained by multiplying the elastic deflection $u_{0}$ of a similar purely elastic beam by the creep function $J\left(t\right)$, which characterizes the specific medium and the instantaneous elastic modulus $E_{0}$.

Equation (23) allows for the determination of the numerical values of the parameters $E_{j}^{i}$ and $\eta_{j}^{i}$ that appear in the creep function $J\left(t\right)$, provided that the deflections $u_{0}$ and $u_{t}$—which can be obtained from creep tests are known. By knowing $u_{0}$ and $u_{t}$, the parameters $E_{j}^{i}$ and $\eta_{j}^{i}$ can be estimated using the least squares method. The basis for the calculations was the averaged deflection values of all the beams.

In each of the analyzed rheological models, there is a parameter $E_{0}$ which represents the Young’s modulus of wood. Its value was determined using the known average experimental deflection at the moment of load application ${\bar{u}}_{0d}$ and the equation:

(24) $$u_{0}=\frac{F\cdot a}{24\cdot E_{0}\cdot I}\cdot \left(3\cdot l^{2}-4\cdot a^{2}\right),$$

which allows for calculating the deflection at the center of the beam span (see Figure 1)—where *l* is the span of the beam, and *a* is the distance from the support to the point of force application. By transforming Equation (24), a formula for calculating the longitudinal elastic modulus was obtained:

(25) $$E_{0}=\frac{F\cdot a}{24\cdot u_{0}\cdot I}\cdot \left(3\cdot l^{2}-4\cdot a^{2}\right),$$

## 3. Results and Discussion

### 3.1. Results of Destructive Testing

**Figure 2:**
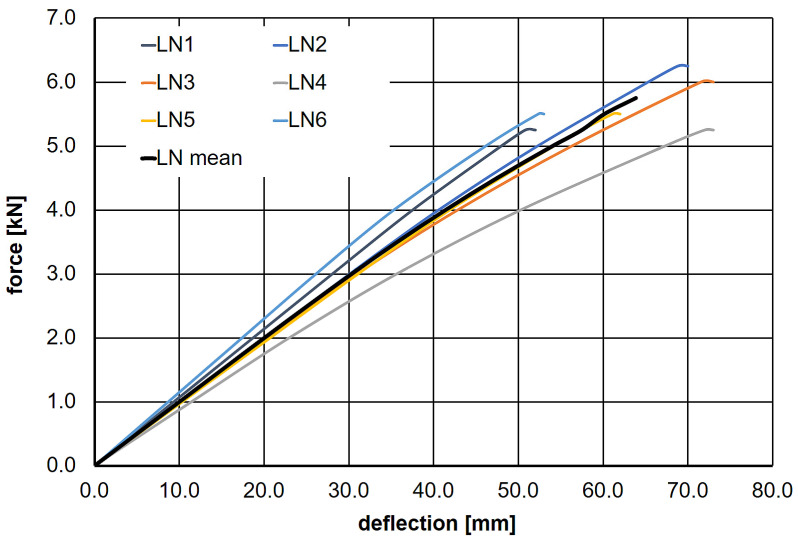
The load–deflection relationships for beams LN1–LN6, along with the average values obtained from the destructive testing program.

As part of the destructive testing program, load–deflection diagrams were generated for the wooden beams. Figure 2 shows the loading curves for all six tested beams (LN1–LN6), along with the average LN mean curve.

Immediately after completing the destructive tests, samples were collected to determine the moisture content of the wood using the oven-drying method. The moisture content values ranged between 7% and 11%, average of 8.8%. The average density was 518.5 kg/m^3^.

The immediate cause of failure in five out of the six tested beams was the presence of knots in the wood. Only in the case of beam LN2 did fiber rupture occur in the tensile zone without the presence of a knot. Throughout almost the entire load range, the load–deflection relationship was linear (Figure 2). The mean failure force was determined to be ${\bar{F}}_{n}^{L}$ = 5.63 kN, with a standard deviation of s = 0.41 kN. Based on this, the average normal stress in the outer fibers of the beam at the moment of failure was calculated  = 49.1 MPa. This result was used to define the load levels for the rheological tests, as described earlier.

### 3.2. Results of Rheological Tests

**Figure 3:**
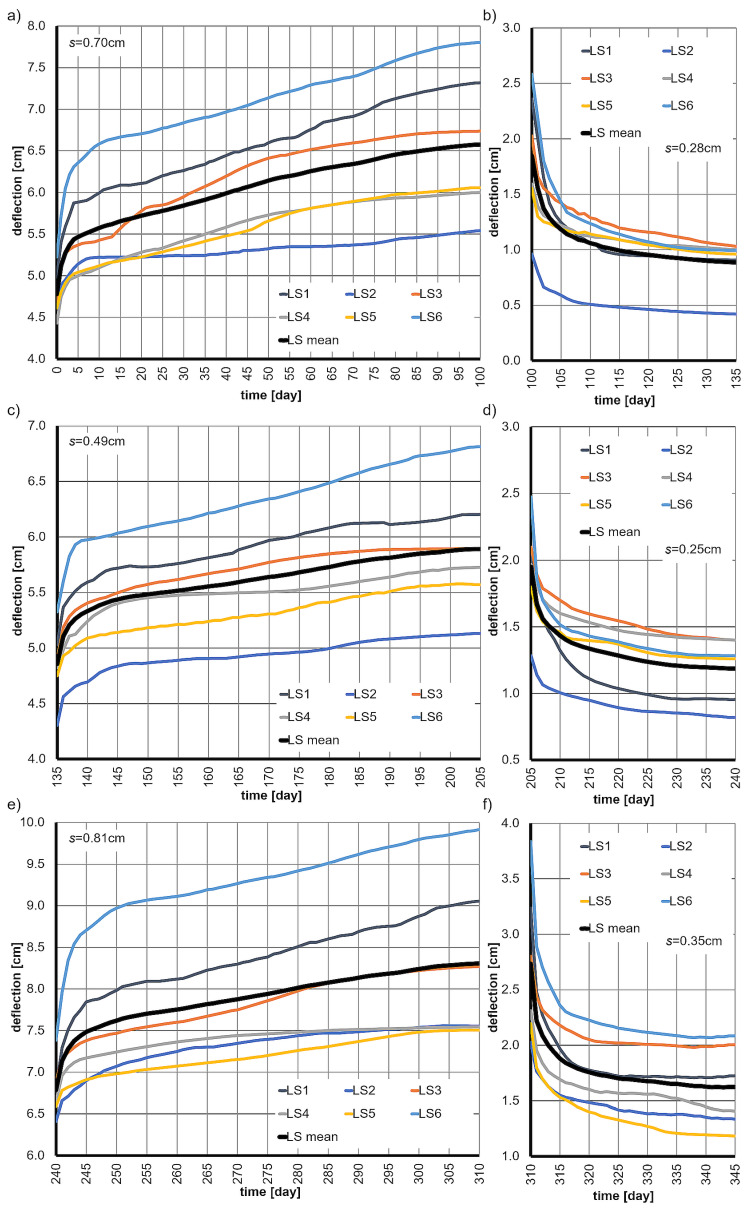
The deflection functions of the beams over time, along with their mean values and average standard deviations *s*, at various stages of loading and unloading are as follows: (**a**) at 30% of the ultimate load, (**b**) unloading from the 30% level, (**c**) at 25% of the ultimate load, (**d**) unloading from the 25% level, (**e**) at 35% of the ultimate load, (**f**) unloading from the 35% level.

The results of the tests, presented as the deflection functions of all six beams, along with the mean deflections and the average standard deviations *s* are shown in Figure 3. It is important to note the significant scatter in the deflection values of the beams (standard deviation *s*), which is associated with the inhomogeneity of the wood and the difficulties in obtaining samples with similar properties.

Using the average elastic deflection *u*_0d_ and Formula (25), the Young’s modulus of the wood was determined to be *E*_0_ = 1.18 × 10^4^ N/mm^2^. This value is common to all the analyzed rheological models.

As a measure of how well the theoretical calculations fit the experimental data, the average relative percentage root mean square deviation was adopted $s_{wo}$:

(26) $$s_{wo}=\sqrt{\frac{1}{l}\cdot {\sum_{k=1}^{l}\left(\frac{u_{tt}\left(x,\;t_{k},\;E_{j},\;\eta_{j}\right)-{\bar{u}}_{td}\left(x,\;t_{k}\right)}{u_{tt}\left(x,\;t_{k},\;E_{j},\;\eta_{j}\right)}\right)}^{2}}\cdot 100\%.$$

For further considerations, the rheological model with the smallest value of $s_{wo}$ was selected. Other measures of fit, such as Pearson’s and Spearman’s correlation coefficients, are not suitable for analyzing data with a nonlinear pattern [35].

### 3.3. Rheological Models and Their Parameters

**Figure 4:**
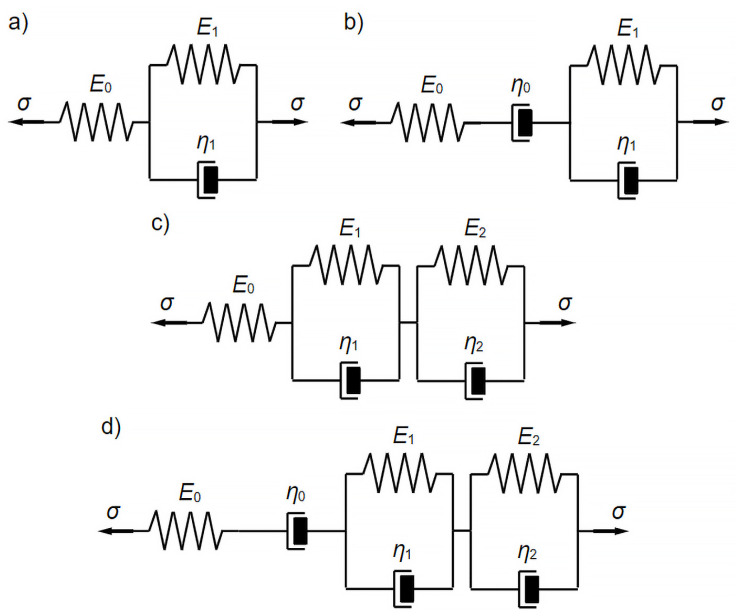
Conceptual schematics of the rheological models used are: (**a**) the three-parameter standard model, (**b**) the four-parameter Burgers’ model, (**c**) the five-parameter model, (**d**) the six-parameter model.

Four linear viscoelastic models, differing in the number and configuration of their constituent elements—represented by Hooke’s (spring) and Newton’s (dashpot) elements were employed to mathematically model the experimentally obtained deflection functions of the wood over time. Schematics of these models are shown in Figure 4.

**Figure 5:**
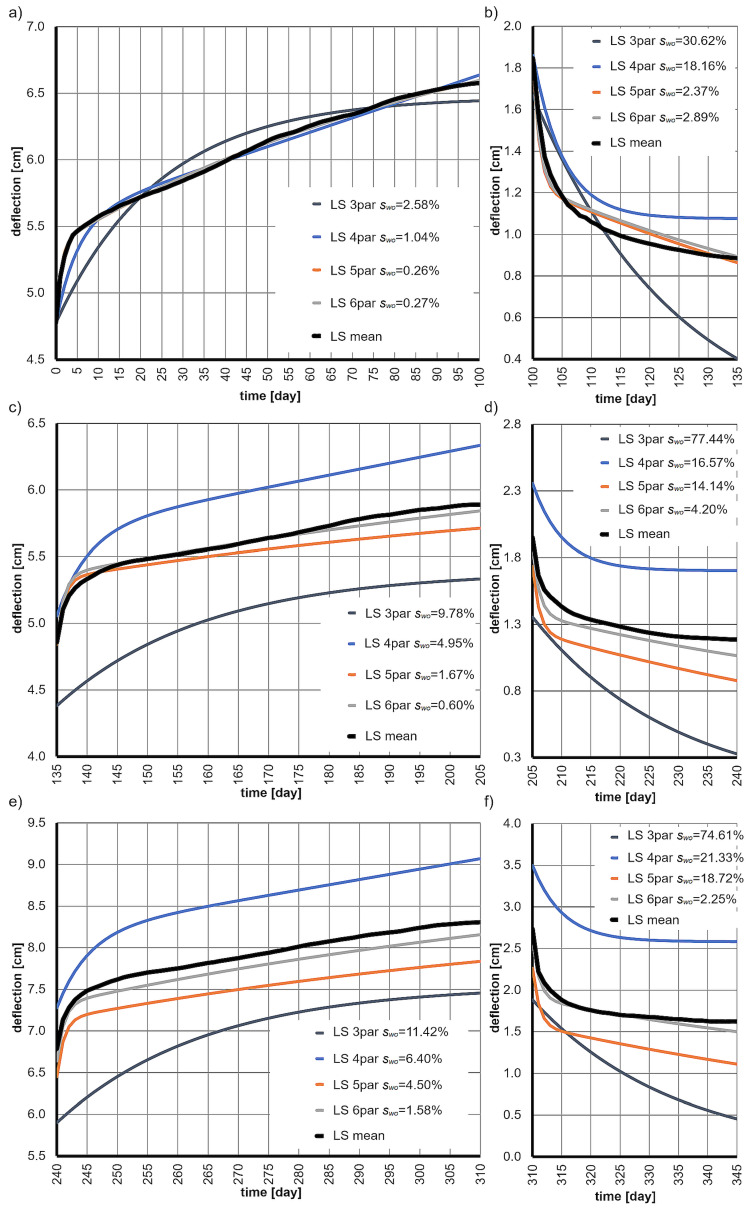
The average deflection functions, creep functions, and schematics of the rheological models used, along with their measures of fit *s_w0_* at various stages of loading and unloading, are presented as follows: (**a**) at 30% of the ultimate load, (**b**) unloading from the 30% load level, (**c**) at 25% of the ultimate load, (**d**) unloading from the 25% load level, (**e**) at 35% of the ultimate load, (**f**) unloading from the 35% load level.

The theoretical deflection curves for these rheological models, along with the experimental mean deflections and their standard deviations (*s*), are presented in Figure 5.

A detailed description of each model and the insights gained from its application are provided in the subsequent subchapters. The numerical values of the parameters for each model, determined using the previously outlined procedure based on the average deflections during the initial load period (0–100 days), are listed in Table 1.

### 3.4. Three-Parameter Rheological Model

The standard three-parameter rheological model, as shown in Figure 4a, was tested first. This model is well described in the literature [30]. Based on this (the creep function of the model) and using relation (23), the formula for the deflection of the viscoelastic beam in this model was derived:

(27) $$u_{tt}=u_{0t}\cdot \left[1+\frac{E_{0}}{E_{1}}\cdot \left(1-e^{-\frac{E_{1}\cdot t}{\eta_{1}}}\right)\right].$$

The obtained results indicate that the three-parameter model is too simple and does not adequately describe the creep behavior of wood. The deviation values $s_{wo}$ are very large at each stage of the tests, and the theoretical function does not align well with the experimental data.

### 3.5. Four-Parameter Rheological Model

The four-parameter Burgers’ rheological model, as shown in Figure 4b, was tested second. This model is also well described in the literature [30]. Based on this (the model’s creep function) and using relation (23), the formula for the deflection of the viscoelastic beam in this model was derived:

(28) $$u_{tt}=u_{0t}\cdot \left[1+\frac{E_{0}\cdot t}{\eta_{0}}+\frac{E_{0}}{E_{1}}\cdot \left(1-e^{-\frac{E_{1}\cdot t}{\eta_{1}}}\right)\right].$$

The conclusions are similar to those of the previous model. The four-parameter Burgers’ model demonstrates significant limitations and is too simplistic to adequately describe the creep behavior of wood.

### 3.6. Five-Parameter Rheological Model

The findings from the current mathematical modeling of wood behavior under long-term loading indicate that relatively simple models of linearly viscoelastic bodies—specifically, the three-parameter standard model and the four-parameter Burgers model—have notable limitations. Consequently, a five-parameter rheological model was implemented, formed by connecting the standard and Kelvin–Voigt models in series. The schematic diagram of this model is shown in Figure 4c.

To determine the numerical values of the parameters $E_{j}^{i}$ and $\eta_{j}^{i}$ in the five-parameter model, it is necessary to know its creep function $J\left(t\right)$. When the component elements are connected in series, their creep functions can be summed [30]. Therefore, the creep function of the model, formed by a series connection of the standard and Kelvin–Voigt models, takes the form:

(29) $$J\left(t\right)=\frac{1}{E_{0}}+\frac{1}{E_{1}}\cdot \left(1-e^{-\frac{E_{1}\cdot t}{\eta_{1}}}\right)+\frac{1}{E_{2}}\cdot \left(1-e^{-\frac{E_{2}\cdot t}{\eta_{2}}}\right).$$

Based on this function (29) and utilizing relation (23), the formula for the deflection of the viscoelastic beam in this model was derived:

(30) $$u_{tt}=u_{0t}\cdot \left[1+\frac{E_{0}}{E_{1}}\cdot \left(1-e^{-\frac{E_{1}\cdot t}{\eta_{1}}}\right)+\frac{E_{0}}{E_{2}}\cdot \left(1-e^{-\frac{E_{2}\cdot t}{\eta_{2}}}\right)\right].$$

Based on the results obtained, it was concluded that the five-parameter model accurately describes wood creep during the initial loading period. An almost perfect agreement with the experimental mean deflection function was achieved. However, during later stages, the theoretical predictions increasingly deviated from the experimental data—particularly during unloading. In the final stage, the deviation value $s_{wo}$ became large and approached that of the simpler four-parameter model. Therefore, the five-parameter model effectively captures the creep behavior of wood under constant, single-step loading, but it is not suitable for multi-stage loading and unloading scenarios.

### 3.7. Six-Parameter Rheological Model

Given the previously unsatisfactory results of the mathematical modeling, a decision was made to use a six-parameter rheological model. This model was developed by connecting the Burgers’ model and the Kelvin–Voigt model in series. The schematic diagram of this model is shown in Figure 4d.

To determine the numerical values of the parameters $E_{j}^{i}$ and $\eta_{j}^{i}$, it is necessary to know the creep function $J\left(t\right)$ of the six-parameter model. This function will be the sum of the creep functions of the Burgers’ model and the Kelvin–Voigt model:

(31) $$J\left(t\right)=\frac{1}{E_{0}}+\frac{t}{\eta_{0}}+\frac{1}{E_{1}}\cdot \left(1-e^{-\frac{E_{1}\cdot t}{\eta_{1}}}\right)+\frac{1}{E_{2}}\cdot \left(1-e^{-\frac{E_{2}\cdot t}{\eta_{2}}}\right).$$

Considering relation (23) and the creep function specified above (31), the deflection of the viscoelastic beam according to this model is expressed as:

(32) $$u_{tt}=u_{0t}\cdot \left[1+\frac{E_{0}\cdot t}{\eta_{0}}+\frac{E_{0}}{E_{1}}\cdot \left(1-e^{-\frac{E_{1}\cdot t_{k}}{\eta_{1}}}\right)+\frac{E_{0}}{E_{2}}\cdot \left(1-e^{-\frac{E_{2}\cdot t_{k}}{\eta_{2}}}\right)\right].$$

Based on the results obtained, it was concluded that the six-parameter model provides an excellent description of wood creep at all stages of loading and unloading. The fit with the experimental mean deflection function was nearly perfect, and the values of the deviations $s_{wo}$ did not exceed 4.20%. It is important to emphasize once again that the parameter values of the model were determined solely based on the experimental results from the first load period (0–100 days). The subsequent stages served only to validate the obtained results.

## 4. Conclusions

Based on the conducted experimental research program and the results of the mathematical modeling of wood creep, the following conclusions can be drawn:

1. Despite its widespread availability and generally favorable mechanical properties, wood exhibits clear rheological characteristics and undergoes significant creep, which is a notable disadvantage in structural applications. In the conducted tests, after 100 days of loading, the average deflection reached 137% of the initial elastic deflection;
2. Simple models of linearly viscoelastic bodies, namely the three-parameter standard model and the four-parameter Burgers’ model, exhibit significant limitations and are too simplistic to adequately describe the creep processes of wood, especially during the initial phase of this phenomenon and under multi-stage loading conditions;
3. Complex models, formed by series connection of the Kelvin–Voigt model to either the standard model or the Burgers’ model, provide a very accurate description of wood rheology under multi-stage loading programs.
4. The ultimately adopted six-parameter model proved to be sufficiently accurate in representing the behavior of wood within the scope of the conducted research program, and simple enough to be used in further work on the rheology of multi-material beams, which will be the subject of future publications. However, due to the numerous factors affecting the performance of wooden structures, this is certainly not the final model. Additionally, the timescale of the conducted tests is too short to draw far-reaching conclusions;
5. The definitive verification of the adopted rheological model can only be ensured through experimental testing employing a multi-stage load program, which clearly separates the process of parameter identification from model validation. Based on the values of the root mean square deviations presented in the previous subsections, it follows that the six-parameter model is significantly better suited for describing wood rheology than the five-parameter model, despite both models showing similar and high agreement with experimental data during the initial loading period used for parameter identification;
6. The root mean square deviation values $s_{wo}$ unloading periods are, for both models, higher than during loading. This observation may indicate, for example, a nonlinear nature of the wood creep process and highlights the need for further research to develop increasingly accurate theoretical models. Based on the obtained test results, especially during unloading periods, it is unfortunately not possible to answer the question of whether permanent viscoplastic deformations exist in the wood. This would require experiments involving very long loading and unloading periods, which is very difficult from a practical standpoint. At the same time, this is a highly interesting scientific problem, which essentially determines the choice of the rheological model (either a linear viscoplastic or nonlinear model). The authors managed to find a linear viscoplastic model that fits the experimental data well, but the 35-day unloading periods were too short for a comprehensive assessment of the applicability of this model in that aspect. Similarly, other researchers’ work does not provide clear answers in this regard;
7. Changes in temperature and humidity conditions can significantly influence rheological processes in wood. In the experiments conducted by the authors and described here, this effect was neglected. It was assumed that temperature and humidity variations in the environment, which are relatively small, do not have a significant impact on the rheological behavior of wooden beams at a full-scale level. However, when developing rheological models of wood for engineering purposes, this influence must not be overlooked.

## Figures and Tables

**Table 1:** Parameter values of the applied rheological models.

Rheological Model	Parameter	Unit	Value
three-parameter rheological model (Figure 4a)	*E*_1_	N/mm^2^	3.31 × 10^4^
	*η*_1_	Ns/mm^2^	7.05 × 10^10^
four-parameter rheological model (Figure 4b)	*η*_0_	Ns/mm^2^	4.51 × 10^11^
	*E*_1_	N/mm^2^	7.17 × 10^4^
	*η*_1_	Ns/mm^2^	3.20 × 10^10^
five-parameter rheological model (Figure 4c)	*E*_1_	N/mm^2^	9.51 × 10^4^
	*η*_1_	Ns/mm^2^	9.28 × 10^9^
	*E*_2_	N/mm^2^	2.89 × 10^4^
	*η*_2_	Ns/mm^2^	2.51 × 10^11^
six-parameter rheological model (Figure 4d)	*η*_0_	Ns/mm^2^	1.32 × 10^12^
	*E*_1_	N/mm^2^	9.56 × 10^4^
	*η*_1_	Ns/mm^2^	9.55 × 10^9^
	*E*_2_	N/mm^2^	4.93 × 10^4^
	*η*_2_	Ns/mm^2^	3.00 × 10^11^

## Data Availability

The original contributions presented in this study are included in the article. Further inquiries can be directed to the corresponding author.
